# A systematic review of implementation strategies for assessment, prevention, and management of ICU delirium and their effect on clinical outcomes

**DOI:** 10.1186/s13054-015-0886-9

**Published:** 2015-04-09

**Authors:** Zoran Trogrlić, Mathieu van der Jagt, Jan Bakker, Michele C Balas, E Wesley Ely, Peter HJ van der Voort, Erwin Ista

**Affiliations:** Department of Intensive Care, Erasmus MC, University Medical Center, P.O. Box 2040, Rotterdam, CA 3000 the Netherlands; College of Nursing, Center of Excellence in Critical and Complex Care, The Ohio State University, Ballantrae Place Dublin Ohio 43016, Columbus, Ohio 6756 USA; Department of Medicine, Division of Pulmonary and Critical Care, Health Services Research Center, Vanderbilt University Medical Center, Nashville, TN 37232 USA; Veteran’s Affairs Tennessee Valley Geriatric Research Education Clinical Center (GRECC), 1215 21st Avenue South MCE Suite 6100, Nashville, TN 37232 USA; Department of Intensive Care, Onze Lieve Vrouwe Gasthuis, P.O. Box 95500, Amsterdam, 1090 HM The Netherlands; Department of Pediatric Surgery, Intensive Care Unit, Erasmus MC-Sophia Children’s Hospital, University Medical Center, P.O. Box 2060, Rotterdam, 3000 CB The Netherlands

## Abstract

**Introduction:**

Despite recommendations from professional societies and patient safety organizations, the majority of ICU patients worldwide are not routinely monitored for delirium, thus preventing timely prevention and management. The purpose of this systematic review is to summarize what types of implementation strategies have been tested to improve ICU clinicians’ ability to effectively assess, prevent and treat delirium and to evaluate the effect of these strategies on clinical outcomes.

**Method:**

We searched PubMed, Embase, PsychINFO, Cochrane and CINAHL (January 2000 and April 2014) for studies on implementation strategies that included delirium-oriented interventions in adult ICU patients. Studies were suitable for inclusion if implementation strategies’ efficacy, in terms of a clinical outcome, or process outcome was described.

**Results:**

We included 21 studies, all including process measures, while 9 reported both process measures and clinical outcomes. Some individual strategies such as “audit and feedback” and “tailored interventions” may be important to establish clinical outcome improvements, but otherwise robust data on effectiveness of specific implementation strategies were scarce. Successful implementation interventions were frequently reported to change process measures, such as improvements in adherence to delirium screening with up to 92%, but relating process measures to outcome changes was generally not possible. In meta-analyses, reduced mortality and ICU length of stay reduction were statistically more likely with implementation programs that employed more (six or more) rather than less implementation strategies and when a framework was used that either integrated current evidence on pain, agitation and delirium management (PAD) or when a strategy of early awakening, breathing, delirium screening and early exercise (ABCDE bundle) was employed. Using implementation strategies aimed at organizational change, next to behavioral change, was also associated with reduced mortality.

**Conclusion:**

Our findings may indicate that multi-component implementation programs with a higher number of strategies targeting ICU delirium assessment, prevention and treatment and integrated within PAD or ABCDE bundle have the potential to improve clinical outcomes. However, prospective confirmation of these findings is needed to inform the most effective implementation practice with regard to integrated delirium management and such research should clearly delineate effective practice change from improvements in clinical outcomes.

**Electronic supplementary material:**

The online version of this article (doi:10.1186/s13054-015-0886-9) contains supplementary material, which is available to authorized users.

## Introduction

*‘*The problem of delirium is far from an academic one. Not only does the presence of delirium often complicate and render more difficult the treatment of a serious illness, but also it carries the serious possibility of permanent irreversible brain damage’, Engel and Romano [1].

This quote, written over 50 years ago by icons in the field of medicine, would seem to be a clarion call for those caring for humans suffering from serious disease. Elsewhere in the same classic manuscript, Engel and Romano make two statements about inadequacies of the approach taken by healthcare professionals in treating delirium: *‘*They seem to have little interest in and, indeed, often completely overlook delirium’ [1,2] and *‘*The deficiencies in the education of many physicians will equip them to recognize any but the most flagrant examples of delirium.’ Even when armed with the wealth of information present in the literature over the past decade about the importance of assessing, preventing and managing delirium in the ICU, effecting the needed changes in care through appropriate implementation programs still requires a substantial change in culture and attention to human factors that are often beyond the scope of training of most medical teams.

In the Society of Critical Care Medicine’s recently released *Clinical Practice Guideline for the Management of Pain, Agitation, and Delirium (PAD) in Adult Patients in the ICU* current evidence is brought together on optimal management of pain, agitation, sedation and delirium [3]. A previously constructed framework to facilitate the implementation of many aspects of the evidence described in the PAD guidelines is the awakening and breathing coordination, choice of sedative, delirium monitoring and management and early mobility (ABCDE) bundle. The ABCDE bundle is specifically aimed at minimizing sedation, encouraging early ventilator liberation, improving delirium assessment and management and facilitating early mobilization in the ICU [4]. Importantly, both the protocols of the trial that established the value of the ABCs [5] and the seminal randomized controlled trial (RCT) that established the positive effects of early mobilization in critically ill patients [6] included routine daily delirium assessments with the Confusion Assessment Method for the Intensive Care Unit (CAM-ICU), with the latter study even establishing a significant reduction in delirium incidence. Therefore, current evidence suggests that: 1) clinical effectiveness of the ABC and E within the ABCDE bundle implies routine delirium assessment with a validated tool, and, inversely, 2) delirium prevention and management requires an integrated multidisciplinary approach with standardized care processes including early mobilization, which in turn is linked to a strategy of minimizing sedation by means of ‘awake(ning) and spontaneous breathing coordination’. As such, ‘brain failure’ (that is, delirium and coma) may be regarded as avoidable and representing an intermediate state on the pathway towards adverse outcomes, such as death and increased length of ICU stay [7]. However, although from the ABCDE bundle or PAD guidelines it may seem evident what to aim for in everyday clinical practice, health care professionals often struggle with how to implement guidelines, especially when these include integrated care covering many domains concurrently and involving multiple care providers.

Therefore, this systematic review of the literature aims at summarizing the implementation strategies and their effectiveness to improve practices of assessment, prevention or management of delirium and clinical outcomes in the critically ill.

## Methods

### Search strategy and selection criteria

This review was performed according to the *Preferred Reporting Items for Systematic Reviews and Meta-Analyses (*PRISMA) guidelines [8]. We searched PubMed, Embase, PsychINFO, Cochrane and CINAHL for studies published between January 2000 and April 2014 with no search filter limits. The year 2000 was chosen because a preliminary Pubmed search with the search terms ‘delirium’, ‘implementation’ and one of ‘ICU’, ‘critically ill’, or ‘critical care’, yielded only one study that year that pertained to the subject of this review and none before [9]. A biomedical information specialist at the medical library of the Erasmus MC - University Medical Center Rotterdam guided the search. Search terms included intensive care and delirium, and were tailored to each database and its indexing system (see Additional file 1). Reference lists of retrieved articles, reviews and books were screened to identify additional papers that met the inclusion criteria.

### Selection of studies

Our search focused on clinical studies aimed at implementation of delirium screening, prevention or management in the adult ICU setting. Implementation could be focused at single components of delirium care (for example, delirium screening) or could include delirium screening, prevention and/or management as an integral part of a wider bundle or guideline (for example, ABCDE bundle or PAD guideline). We considered the PAD guideline and the ABCDE bundle as similar for the purpose of this review because, next to delirium screening, they share several integrated evidence-based care components (early mobilization, awakening and breathing coordination or targeting light sedation and systematic pain assessment and treatment). We did not limit the search to specific types of ICU. To be included in the review, the study had to contain a clear description of the implementation process (that is, an explanation of what exactly was done to implement it). We excluded studies that concerned delirium related to alcohol withdrawal and/or were focused solely on validation of delirium screening tools. Further, the efficacy of the implementation intervention had to be reported in terms of a clearly defined outcome such as mortality, length of stay, and/or adherence to delirium screening. Reviews, opinion papers, editorials and comments on original articles were also excluded.

Two authors (ZT, EI) independently checked abstracts of retrieved articles on compliance with selection criteria. Relevant full-text articles were checked for final inclusion. Consensus on final selection was achieved by discussion with a third author (MJ).

### Data extraction and synthesis

The first reviewer (ZT) extracted data on design, population, implementation strategies, and outcomes and studies were subject to further critical appraisal by two other authors (EI, MJ). The individual implementation strategies were classified into four categories: professional (for example, distribution of educational materials, reminders), organizational (for example, provider-oriented interventions, structural interventions), financial and regulatory (for example, peer review, changes in medical liability) using the *Cochrane Effective Practice and Organization of Care* group (EPOC) classification system checklist (Table 1) [10]. From these 4 categories, we then distinguished 17 individual implementation strategies (Table 1). The implementation strategies concern all phases of a formal implementation process as has been described before in the literature [11]. For instance, the strategy of ‘marketing/tailored interventions’ includes first performing an analysis of barriers to implementation to be able to design a subsequent implementation strategy addressing these barriers, to enhance implementation effectiveness. As such, the use of more strategies concurrently may indicate a more complete implementation process.

**Table 1:** **Implementation strategy taxonomy according to the EPOC classification system**

**Category**	**Individual strategies**	**Description**
Professional	1. Distribution of educational materials	Distribution of published or printed recommendations for clinical care, including clinical practice guidelines, audio-visual materials and electronic publications. The materials may have been delivered personally or through mass mailings.
	2. Educational meetings	Conferences, lectures, workshops or traineeships.
	3. Local consensus processes	Inclusion of participating providers in discussion to ensure that they agreed that the chosen clinical problem was important and the approach to managing the problem was appropriate.
	4. Outreach visits	Use of a trained person who met with providers in their practice settings to give information with the intent of changing the provider’s practice. The information given may have included feedback on the performance of the provider(s).
	5. Local opinion leader	Use of providers nominated and explicitly identified by their colleagues as educationally influential.
	6. Patient-mediated intervention	New, previously unavailable clinical information collected directly from patients and given to the provider; for example, patient depression scores from a survey instrument.
	7. Audit and feedback	Any summary of clinical performance of health care over a specified period of time. The summary may also have included recommendations for clinical action. The information may have been obtained from medical records, computerized databases, or observations from patients.
	8. Reminders	Patient or encounter-specific information, provided verbally, on paper or on a computer screen, which is designed or intended to prompt a health professional to recall information. This would usually be encountered through their general education; in the medical records or through interactions with peers, and so remind them to perform or avoid some action to aid individual patient care. Computer-aided decision support and drugs dosage are included.
	9. Marketing / Tailored interventions	Use of personal interviewing, group discussion (focus groups), or a survey of targeted providers to identify barriers to change and subsequent design of an intervention that addresses identified barriers.
	10. Mass media	(1) Varied use of communication that reached great numbers of people including television, radio, newspapers, posters, leaflets, and booklets, alone or in conjunction with other interventions; (2) targeted at the population level.
Organizational	11. Provider oriented interventions	Revision of professional roles, for example, expansion of role to include new tasks; creation of clinical multidisciplinary teams who work together; formal integration of services; skill mix changes (changes in numbers, types or qualifications of staff); arrangements for follow up; satisfaction of providers with the conditions of work and the material and psychic rewards (for example, interventions to boost morale); communication and case discussion between distant health professionals
	12. Patient oriented interventions	Mail order pharmacies (for example, compared to traditional pharmacies); presence and functioning of adequate mechanisms for dealing with patients’ suggestions and complaints; consumer participation in governance of health care organization; other categories
	13. Structural interventions	Changes to the setting/site of service delivery; changes in physical structure, facilities and equipment; changes in medical records systems (for example, changing from paper to computerized records); changes in scope and nature of benefits and services; presence and organization of quality monitoring mechanisms; ownership, accreditation, and affiliation status of hospitals and other facilities; staff organization
Financial	14. Provider or patient interventions	In summary: patient or provider is financially supported to execute specific actions. For detailed definitions, see reference [10]
Regulatory	15. Changes in medical liability	Any intervention that aims to change health services delivery or costs by regulation or law (these interventions may overlap with organizational and financial interventions).
	16. Management of patient complaints	
	17. Peer review or Licensure	

EPOC, Cochrane Effective Practice and Organisation of Care group.

With regard to the outcomes, we distinguished between clinical outcomes (ICU length of stay (LOS) and mortality) and process outcomes (adherence to screening for the presence of delirium, knowledge of delirium, incidence of delirium, use of antipsychotics) [12]. Changes in these outcomes were assessed before and after implementation (or with and without implementation in the case of the only RCT included). Three authors (EI, ZT, MJ) independently scored the implementation strategies in the implementation studies reporting clinical outcomes. Differences in assessment were resolved afterwards by discussion. The studies that did not report mortality were assessed equally by two authors (ZT, MJ). We tabulated the key features deemed important for this review of all included studies: number and type of implementation strategies, care components (that is, using integrated strategy such as PAD/ABCDE or separate interventions such as screening only), implementation model and the process and clinical outcomes as previously defined.

### Methodological quality

We rated the methodological quality of all implementation studies in an effort to ascertain a minimum quality of included studies. We used a rating system adapted from Anderson and Sharpe [13] (see Additional file 2), which evaluated the impact of various types of intervention on the behavior change of health care workers, in line with our review. Two reviewers (ZT/EI) independently assessed each study on quality and differences in quality scores were resolved through discussion. Studies that rated fewer than three points were excluded because of very poor methodological quality.

### Statistical analyses

Associations between study characteristics and outcomes were assessed with Pearson’s chi-square or Fisher’s exact test after dichotomization (for example, significant decrease of delirium incidence: yes/no). The number of implementation strategies used in the implementation studies was summarized as median with IQR.

Whenever possible, for meta-analysis we quantitatively pooled the results at the patient level for the included studies when the original data were retrievable. We contacted the authors of the original articles for these data when not provided in the published paper. We expressed the effectiveness of the implementation interventions as a risk ratio (RR) for dichotomous outcomes by using a DerSimonian and Laird random-effect model [14] and as a weighted mean difference (WMD) for continuous outcomes with 95% CIs. The heterogeneity among studies was tested using the Cochran *Q*-test of heterogeneity, and Higgins and Thompson *I*^*2*^ [15]. The degree of heterogeneity was defined as a value of *I*^*2*^: low (25% to 49%), moderate (50% to 74%), and high (>75%) values [15]. Subgroup analysis was performed for number of implementation strategies (low number = below median, high number = median or higher), and use of either PAD guideline/ABCDE bundle. Analysis was performed with Microsoft Excel 2013 and IBM SPSS 21.0. Statistical significance was defined as a *P*-value <0.05.

## Results

### Selection of studies

**Figure 1:**
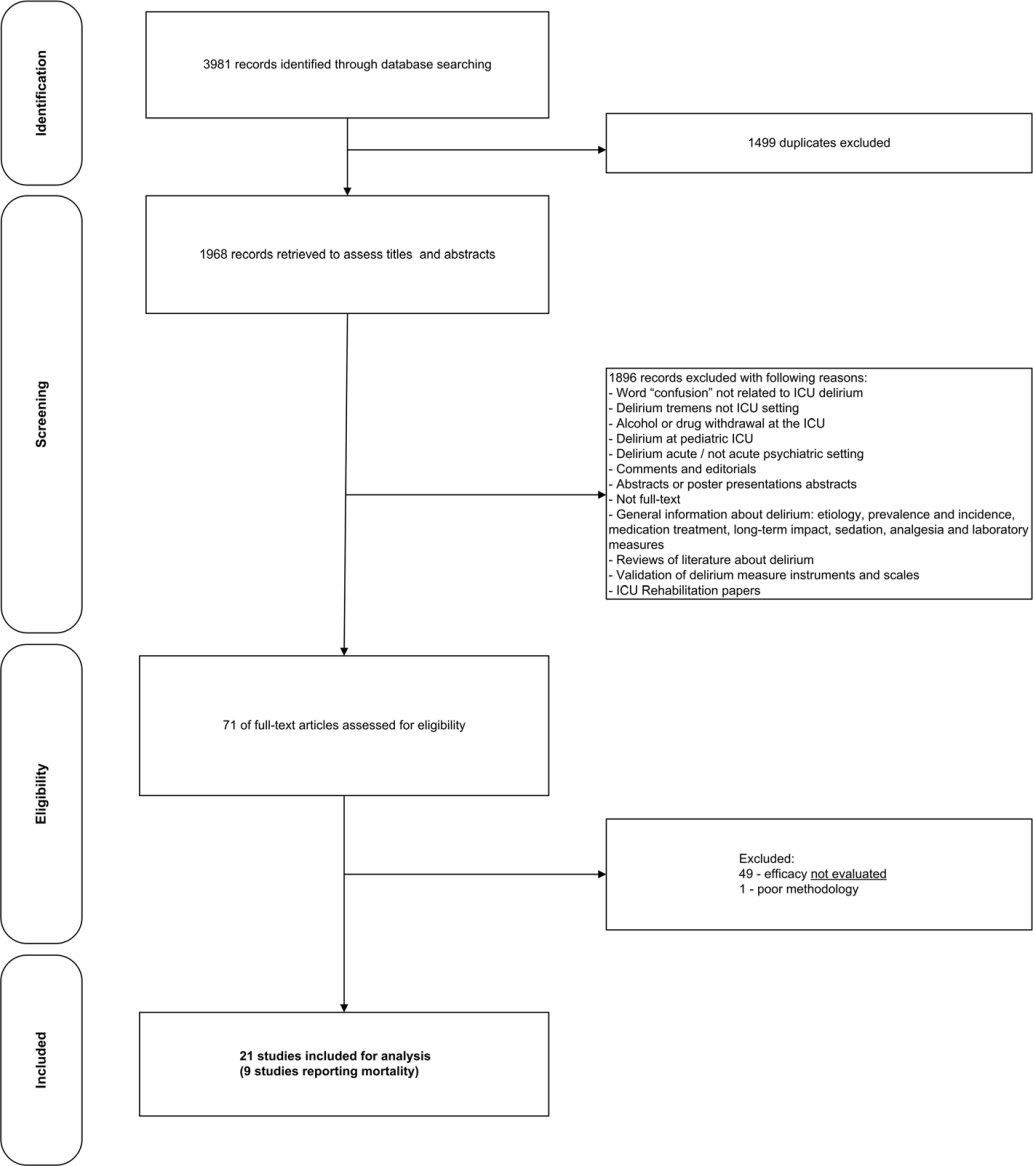
**Selection of included studies for the review.**

We reviewed 3,981 hits and after excluding duplicates and studies not meeting inclusion/exclusion criteria, 21 studies were evaluated [16-36] (Figure 1). Mortality and ICU-LOS changes were reported in ten studies [16,20,24,26-28,30,32,35,36] and in one study ICU-LOS was reported but not mortality [33]. One publication was a duplicate with regard to study period and population and was therefore excluded from the analysis of clinical outcomes but included in the assessment of studies that reported process measures [27]. Sixteen of twenty-one included studies were before-after studies; one was an RCT, and the remaining studies were prospective or retrospective cohort studies.

### Methodological quality

One study was of very low methodological quality (2 points) and was excluded [37] (see Additional file 3: methodological quality rating of included studies and Figure 1). This study was a randomized trial but details on randomization, interventions and assessment of delirium were insufficient with regard to reproducibility.

### Implementation strategies

Implementation strategies that were used in the 21 included studies reporting process and clinical outcomes are shown in Table 2 (strategies are explained in Table 1). These studies were published between 2005 and 2014. Professional-oriented strategies (that is aimed at changing professionals’ behavior) and organizational strategies (that is aimed at changing the structure of care delivery) were the most frequently used categories of implementation strategies. Of the professional-oriented strategies, education (meaning one or both of the following strategies: distribution of educational material (81%) and/or educational meetings (100%), was used in all studies (Tables 1 and 2). Patient-mediated interventions, corresponding with implementation of screening for delirium with a validated tool such as CAM-ICU, was applied in 86% of the studies, whereas outreach visits, audit and feedback and local consensus processes were applied in 67%, 62% and 57% of the studies respectively (Table 2). Three of the seventeen implementation strategies were not used at all (that is mass media, changes in medical liability and management of patient complaints). Three strategies were used in only one or two studies (provider-oriented interventions/financial compensation [24], licensure [16] and patient-oriented interventions [16,31]). Tailored interventions were used in 33% of the studies [16,20].

**Table 2:** **Summary of implementation strategies**

**Implementation strategy**			**Studies reporting both clinical outcomes and process outcomes before versus after implementation**									Studies reporting process outcomes, without clinical outcomes, before versus after implementation*												**Percent using strategy**
		**Author**	**Mansouri**	**Skrobik**	**Balas**	**Radtke**	**Robinson**	**Kamdar**	**Reade**	**Dale**	**Bryckz.**	**Eastwood**	**Devlin**	**Scott**	**Gesin**	**Riekerk**	**Kastrup**	**Boogaard**	**Pun**	**Hager**	**Soja**	**Page**	**Bowen**	
**PO**	1	Distribution**	1	1	1	1	1	1	0	1	0	0	0	1	1	1	1	1	1	1	1	1	1	81
	2	Educational Meetings	1	1	1	1	1	1	1	1	1	1	1	1	1	1	1	1	1	1	1	1	1	100
	3	Local consensus	1	1	1	0	1	1	0	1	1	0	0	0	1	1	1	1	0	1	0	0	1	62
	4	Outreach	0	0	1	1	0	0	1	1	0	1	1	1	1	1	0	1	1	0	1	1	1	67
	5	Opinion leaders	0	1	1	1	1	0	1	0	0	0	1	0	0	1	0	1	0	1	1	0	1	52
	6	Patient-mediated	1	1	1	1	1	1	1	1	0	1	1	1	1	1	1	1	0	1	1	1	0	86
	7	Audit/feedback	1	1	1	0	0	0	0	0	0	1	1	0	1	1	1	1	1	1	1	0	1	62
	8	Reminders	0	0	1	1	0	1	0	0	0	0	0	0	0	0	1	1	0	1	1	0	1	38
	9	Tailoring (barriers)	0	1	1	0	0	0	0	0	0	0	0	0	0	1	0	1	0	1	1	0	1	33
	10	Mass media	0	0	0	0	0	0	0	0	0	0	0	0	0	0	0	0	0	0	0	0	0	0
**O**	11	Provider-oriented	1	0	1	0	0	1	0	0	0	0	1	0	0	1	0	1	1	1	1	0	0	43
	12	Patient-oriented	0	1	0	0	0	0	0	0	1	0	0	0	0	0	0	0	0	0	0	0	0	10
	13	Structural	0	1	1	1	0	0	0	0	0	0	0	0	1	1	1	1	1	1	1	0	0	48
**F**	14	Provider	1	0	0	0	0	0	0	0	0	0	0	0	0	0	0	1	0	0	0	0	0	10
**R**	15	Medical liability	0	0	0	0	0	0	0	0	0	0	0	0	0	0	0	0	0	0	0	0	0	0
	16	Patient complaints	0	0	0	0	0	0	0	0	0	0	0	0	0	0	0	0	0	0	0	0	0	0
	17	peer review/licensure	0	0	1	0	0	0	0	0	0	0	0	0	0	0	0	0	0	0	0	0	0	5
**Total number IS used**			**7**	**9**	**12**	**7**	**5**	**6**	**4**	**5**	**3**	**4**	**6**	**4**	**7**	**10**	**7**	**12**	**6**	**10**	**10**	**4**	**8**	
Post-implementation***																								
**Mortality**			**⬇**	⬇	⬇	↓	↓	↓	↑	=	↓	↑	-	-	-	-	-	-	-	-	-	-	-	
**ICU length of stay**			⬇	⬇	↓	⬇	↓	↓	=	⬇	⬇	=	-	-	-	-	-	↓	-	-	-	-	-	
**Screening adherence**			⬆	↑	⬆	⬆	-	-	-	⬆	-	-	⬆	⬆	-	⬆	⬆	⬆	⬆	=	⬆	⬆	⬆	
**Incidence**			-	↓	⬇	-	-	⬇	⬇	⬇	↑	-	-	-	-	-	↑	⬆	-	⬆	-	-	-	
**Antypsychotic drug use**			↓	↑	↑	-	↑	-	-	⬇	↓	⬇	-	-	-	-	-	⬇	-	-	-	-	-	
**Delirium knowledge**			-	-	-	-	-	-	-	-	-	-	-	⬆	⬆	⬆	-	⬆	-	-	-	-	-	

*Study by Eastwood concerns the same study population as the study by Reade and was therefore not used for analysis of clinical outcomes. **For explanation of individual strategies, see Table 1.

***Only statistically significant changes are in bold text. PO, professional-oriented; O, organizational; F, financial; R, regulatory; IS, implementation strategies.

### Implementation characteristics, process outcomes and clinical outcomes

The number of implementation strategies used varied from 4 to 12 per study (Table 3). The overall median number of implementation strategies used per study was 7.0 (IQR 4.5 to 9.5). In the studies reporting clinical outcomes (n = 9) versus only process outcomes (n = 12) the median number of used strategies was 6.0 (IQR 4.5 to 8.0) and 7.0 (IQR 7.5 to 10.0) respectively (*P* = 0.46) (Table 2). Within the nine studies with clinical outcomes, the following implementation strategies were reported only in studies with significant mortality reduction (that is, the studies by Mansouri, Skrobik, Balas): tailoring, encouragement for implementation by means of financial incentives, licensure, and audit and feedback (Table 2). Audit and feedback was used in all studies showing significant mortality reduction but in none without significant reduction of mortality (*P* = 0.012). In contrast, these and other strategies were used frequently in studies that reported process outcomes without clinical outcomes. The number of strategies per study belonging to the domains of organizational, financial or regulatory implementation strategies (that is, not aimed at the professional) (Table 2) in the clinical outcome versus the process outcome studies did not differ (*P* = 0.92). However, within the nine clinical outcome studies the studies with significant mortality reduction after the implementation [14,24,31] used more of these non professional-oriented strategies (median 2, IQR 2 to 3) than studies without a significantly reduced mortality [26,28,30,32,35,36] (median 0.5, IQR 0.0 to 1.0, *P* = 0.024).

**Table 3:** **Implementation characteristics and changes in important process and clinical outcomes before versus after implementation**

**Author, year (design)**	**Implementation**			**Process outcomes**				**Clinical outcomes**	
	**Number of strategies used**	**Implemented care components**	**Implementation model**	**Screening adherence**	**Delirium incidence**	**Use of antipsychotic drugs**	**Delirium knowledge**	**Mortality change**	**ICU length of stay, days**
Balas, 2014 [16] (B/A^a^ study, n = 296)	12	ABCDE^b^	CFIR^c^	**+50% (0 to 50%)** ^**d**^	**−13% (62 to 49%),** ***P =*** **0.02**	+12 mg (6 to 18 mg)^e^, *P* = 0.24	-	**−8.6% (19.9 to 11.3%),** ***P*** **= 0.04**	*−1* ^f^ (5 to 4), *P* = 0.21
Van den Boogaard, 2009 [17] (B/A study, n = 1742)	12	Delirium screening	Model of Grol and Wensing	**+14% (77 to 92%),** ***P*** **<0.0001**	**+13% (10 to 23%),** ***P*** **<0.05** ^**g**^	**−12 mg (18 to 6 mg)** ^**e**^ **,** ***P*** **= 0.01**	**+1.2 (6.2 to 7.4),** ***P*** **<0.001**	*-*	*−*0.3 (1.3 to 1)^f^ *P* ***<*** *0.05*
Riekerk, 2009 [18] (B/A study, n = NA)	10	Delirium screening	Structural implementation pathway	**+57% (38 to 95%)** ^**d**^	**-**	**-**	**+1** ^**d,h**^ **(3–4)**	*-*	*-*
Hager, 2013 [19] (B/A study, n = 202)	10	PAD^w^	4Es framework^i^	0 (90 to 90%)	**+18%** ^**j**^ **(20 to 38%),** ***P*** **= 0.01**	**-**	-	*-*	*-*
Skrobik, 2010 [20] (B/A study, n = 1133)	9	PAD	-	+3^k^ (89 to 92%), *P* = 0.055	−0.5% (34.7 to 34.2%), *P* = 0.9	+0.3% (39.4 to 39.7%), *P* = 0.7	-	**−6.5% (29.4 to 22.9%),** ***P*** **= 0.009**	**−0.97** ^**l**^ **(6.32 to 5.35),** ***P*** **= 0.009**
Bowen, 2012 [21] (pilot study, n = 34 nurses)	8	Delirium screening	Diffusion of Innovations theory	**+75% (10% to 85%)**	**-**	**-**	-	**-**	**-**
Soja, 2008 [22] (Prospective study, n = 347)	10	Delirium screening	-	**+84% (0 to 84)** ^**d**^	-	-	-	**-**	**-**
Gesin, 2012 [23] (B/A study, n = 20 nurses)	7	Delirium screening	-	-	**-**	**-**	**+2.1 (6.1 to 8.2),** ***P*** **= 0.001**	**-**	**-**
Mansouri, 2013 [24] (RCT, n = 201)	7	PAD	-	**+100%** ^**m**^ **(0 to 100%)**	-	−2.5 mg^n^ (3.2 to 0.7 mg), *P* = 0.12	-	**−12% (24 to 13%),** ***P*** **= 0.046**	**−3.1 (7.1 to 4.0)** ^**f**^ **,** ***P*** **<0.001**
Pun, 2005 [25] (Prospective study, n = 711)	6	PAD	-	**+90% (0 to 90)** ^**d**^ **+84% (0 to 84)** ^**d**^	**-**	-	-	**-**	**-**
Radtke, 2012 [26] (B/A^e^ study, n = 131)	7	PAD	Modified extended training	**+1.6 (0 to 1.6),** ***P*** **<0.01**	-	-	-	−4.8%^o^ (9.9 to 5.1%), *P* = 0.16	−4 (18 to 14)^p^, *P =* 0.40
									**−4 (8 to 4)** ^**p**^ **,** ***P*** **<0.01**
Eastwood, 2012 [27] (B/A study, n = 288 patients/2368 shifts)	4	Delirium screening	-	**-**	-	**+8.5%** ^**q**^ **(5.8 to 14.3%),** ***P*** **<0.0001** ^**r**^	-	+3.2% (5 to 8.2)^s,t^ *P* = 0.31	0 (2 to 2), *P* = 0.34
Kamdar, 2013 [28] (B/A study, n = 285)	6	Multifaceted sleep promotion program	Structured QI model	-	**odds ratio 0.46** ^**a**^ **,** ***P*** **= 0.02**	**-**	-	−6% (25 to 19%), *P* = 0.88^s^	−1.1^u^ (5.4 to 4.3), *P* = 0.60
Scott, 2012 [29] (B/A study, n = 119)	4	Delirium screening	-	**+78% (0 to 78%)** ^**d**^	**-**	**-**	**+14%** ^**v**^ **(71 to 85%),** ***P*** **<0.001**	**-**	**-**
Dale, 2014 [30] (B/A study, n = 1483)	5	PAD	-	**+1.14** ^**x**^ **(0.35 to 1.49),** ***P*** **<0.01**	**odds ratio 0.67,** ***p*** **= 0.01**	**−1.7 (2.7 to 1.0)** ^**y**^ **,** ***P*** **<0.01**	-	0 (14 to 14%), *P* = 1.0	**−12.4%** ^**j**^ **,** ***P*** **= 0.04**
Kastrup, 2011 [31] (B/A study, n = 205)	7	Visual feedback system	-	**+37.5% (0.5 to 38%),** ***P*** **<0.01**	+4% (25 to 29%), *P* = 1.0^za^	**-**	-	**-**	**-**
Robinson, 2008 [32] (B/A study, n = 119)	5	PAD	-	-	-	+14% (31 to 45%), *P* = 0.25	-	−2.9% (17.6 to 14.7), *P* = 0.64	−1.8 (5.9 to 4.1), *P* = 0.21
Devlin, 2008 [33] (B/A study, n = 601)	6	Delirium screening	SCT^zb^	**+70% (12 to 82%),** ***P*** **<0.0005**	-	**-**	-	-	-
Page, 2009 [34] (Retrospective study, n = 60)	4	Delirium screening	-	**+92% (0 to 92%)** ^**d**^	**-**	-	-	-	-
Reade, 2011 [35] (B/A study, n = 288)	4	Delirium screening	-	-	**−16% (37 to 21%),** ***P*** **= 0.004**	**-**	-	+3.2% (5 to 8.2)^zc^, *P* = 0.31	0 (2 to 2), *P* = 0.34
Bryczkowski, 2014 [36] (B/A study, n = 123)	3	Delirium prevention program	-	-	+11% (58 to 47%), *P* = 0.26	−1% (7 to 6%), *P* = 0.83	-	−4% (7 to 3%), *P* = 0.31	**−3 (9 to 6),** ***P*** **= 0.04**

^a^B/A = before-after. ^b^ABCDE = awakening and breathing coordination, delirium monitoring/management and early exercise/mobilization bundle. ^c^CFIR = Consolidated Framework for Implementation Research. ^d^Statistical significance not reported or assessable from data in article but presumed to be statistically significant because of strong effect (difference before-after shown in parentheses). Significant changes are shown in bold letters. ^e^Total dose of haloperidol per patient. ^f^Median. ^g^Chi-square test. ^h^Increase in median level of agreement on a scale of 5 (1 = totally disagree, 5 = totally agree, with 3 = neutral about statement and 4 = agree) with true statements about delirium, signifying increased knowledge. ^i^4Es framework = Engage, Educate, Execute and Evaluate. ^j^Percent of ICU days delirium present per patient. ^k^Adherence calculated by dividing delirium assessments judged to be possible by total number of patients in Table 1 in reference. Adherence data to screening not explicitly provided in text. ^l^Mean. ^m^No explicit mention of screening adherence, but after CAM-ICU implementation as part of the PAD guideline the authors mention strict adherence surveillance to the PAD protocol: 15 patients in protocol group excluded from analysis because of noncompliance with PAD guideline. ^n^Mean dose of drug (haloperidol) used per patient. ^o^Mortality calculated from numbers given in Table 1 in original article for combined data of ICU 1 and 2 (n = 131, before-after comparison made with chi-squared test, degrees of freedom (df) = 2). ^p^This study reported different interventions (standard training versus extended training and implementation) in different ICUs. Numbers given here are those from the B/A study in two ICUs that received modified extended training. ^q^Percentage is total number of administered doses of either haloperidol (5 mg), olanzapine (5 to 10 mg) or quetiapine (25 mg) divided by the total number of 8-hour shifts in pre- and post-CAM-ICU implementation period. Study of Eastwood is duplicate report of study by Reade, therefore, data were combined for analysis. ^r^Chi-squared statistic = 47, df = 1. ^s^Unstructured delirium screening versus CAM-ICU screening. ^t^Data on change in mortality were not included for analysis of all mortality data because these data are same as those of Reade, 2011 [35]*.* ^u^Calculated for survivors, median, frequency of delirium monitoring per day per patient. ^v^Calculated agreement with true statements about delirium and its importance increased with 14% after the implementation, signifying increased knowledge (chi-squared statistic = 14, df = 1). ^w^PAD = integrated pain, agitation/sedation and delirium monitoring and management; ^x^Number of CAM-ICU assessments/day (mean). ^y^Mean daily haloperidol dose (mg). ^za^Fisher’s exact test. ^zb^SCT = script concordance theory. ^zc^Percent patients ever receiving haloperidol.

Delirium screening adherence was assessed in 15 of the 21 studies, of which 13 showed a significantly increased adherence (Table 3) [16-18,21,22,24-26,29-31,33,34]. In studies specifically focused on implementation of delirium screening (n = 10), improvements in adherence to screening ranged from 14% to 92%, but the definition of adherence varied widely. These studies with focus on delirium screening typically did not report clinical outcomes (1 of 10 studies), whereas process outcomes were assessed in all of these studies (Tables 2 and 3). Significant improvement of screening adherence after the implementation was reported in 82% (9/11) of the studies that did not report on clinical outcomes, versus 56% (5/9) of the studies that assessed clinical outcomes. Use of integrated delirium management (PAD/ABCDE) was reported in 18% (2/11) of studies without clinical outcome assessment versus in 67% (6/9) of studies with clinical outcome. Knowledge improvement was reported in 4 of 21 studies and varied both in magnitude and definition [17,18,23,29]. Knowledge improvement was reported in 36% of studies (4/11) without clinical outcome data, versus 0% in studies with only process outcome data. Changes in reported delirium incidence [16,17,19,20,28,30,31,35,36] and use of antipsychotic drugs [16,17,20,24,27,30,32,36] after implementation varied between studies (some showed increased and some showed decreased incidence, Table 3). No significant associations existed between changes in the process measures (delirium incidence, use of antipsychotic drugs or screening adherence) and mortality before and after the implementation. Likewise, no significant associations were found between the process measures and ICU LOS.

In pooled analysis, we did not find differences in delirium incidence (n = 8) before versus after the implementation when comparing the studies using PAD/ABCDE versus those that did not use these frameworks, or comparing those with high versus low number of implementation strategies, and high inconsistency existed in such pooled analyses (see Additional file 4: Figures S4a,b). Implementation studies focusing on delirium screening tools did not report increased delirium incidence after the implementation compared with studies that used other frameworks (for example, PAD/ABCDE, that is, a more integrated program, see Additional file 4: Figure S4c). Pooled analysis of relations between implementation strategies and adherence rates for screening or knowledge were not possible due to highly variable definitions for the process outcomes, and irretrievable original data.

### ICU-LOS

**Figure 2:**
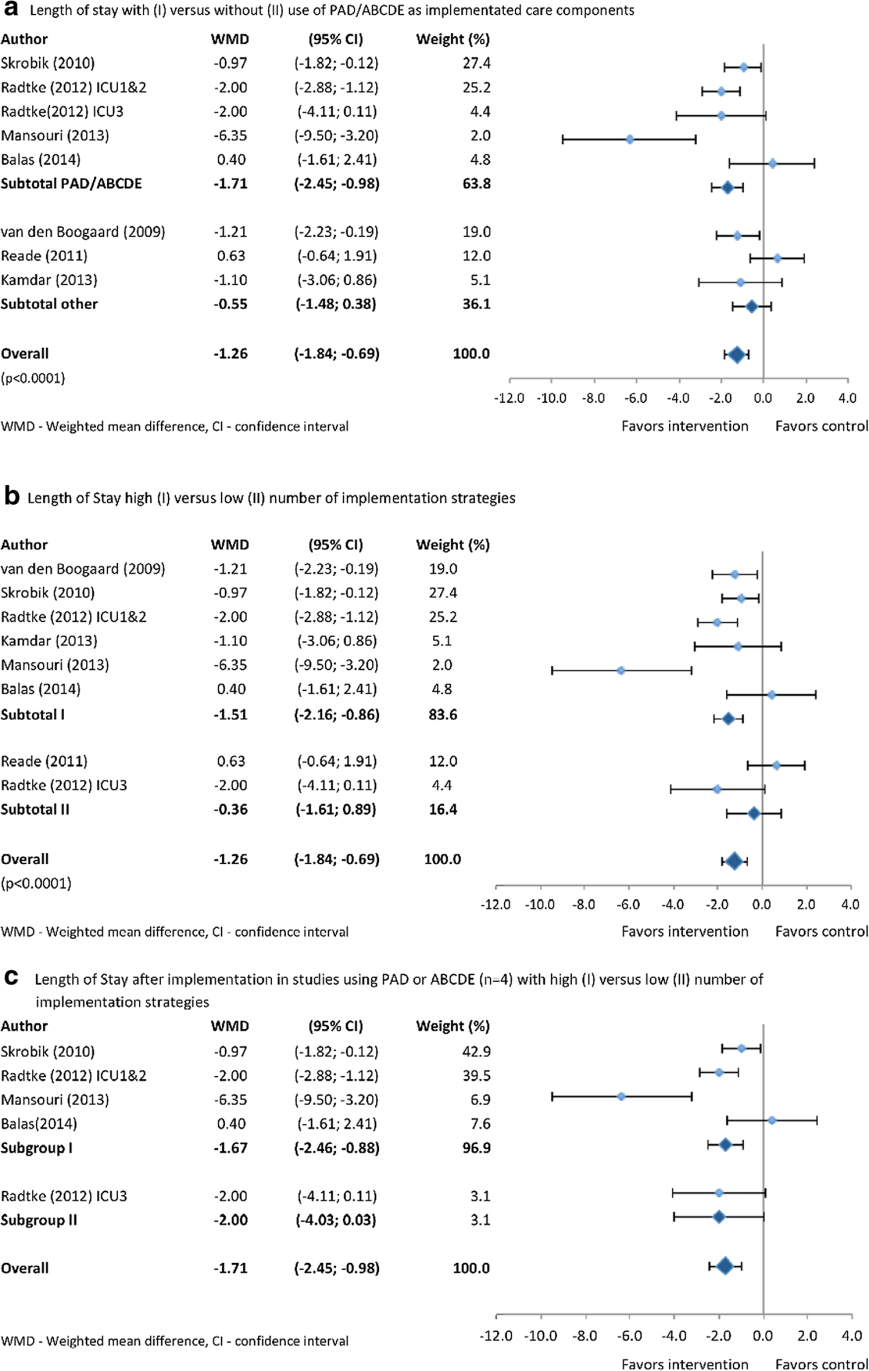
**Pooled analysis of determinants of changes in ICU length of stay (days) in implementation studies (n = 7) that included delirium-oriented interventions.** Determinants of ICU length of stay reduction that were studied were: use of either the guideline for the management of pain, agitation and delirium (PAD) or the awakening and breathing coordination, choice of sedative, delirium monitoring and management and early mobility (ABCDE) bundle **(a)** or use of high or low number of implementation strategies **(b)**. **(c)** Impact of high or low number of strategies within the studies reporting ICU length of stay and using PAD/ABCDE (n = 4). See text for more details. Study by Radtke reported multiple populations and these were separately assessed.

Nine of the ten studies that reported LOS showed reduced ICU LOS after implementation (the study by Eastwood and Reade were the same population); of which five were statistically significant (Tables 2 and 3). The study by Radtke *et al*. [26] included populations from three different ICUs and were stratified according to standard or more intensive implementation strategies. Pooling all studies that reported ICU LOS and of which data were retrieved (n = 7) showed a reduction of −1.26 days (95% CI −1.84, −0.69) after the implementation (Figure 2a). Pooled data from four studies reporting ICU LOS after implementation of PAD or the ABCDE bundle approach yielded significantly shorter LOS after implementation compared with not using these approaches (WMD = −1.71; 95% CI −2.45, −0.98 versus WMD −0.55, 95% CI −1.48, 0.38) (Figure 2a). Using a high (≥6) number of strategies showed a reduced ICU LOS (−1.51, 95% CI −2.16, −0.86) versus no change when using fewer strategies (−0.36, 95% CI −1.61, 0.89) (Figure 2b). Within the studies using PAD or ABCDE (n = 4) the signal that using more strategies reduced ICU LOS was less evident (Figure 2c). None of the individual strategies were used more often in studies with versus without statistically significant ICU LOS reduction.

### Mortality

**Figure 3:**
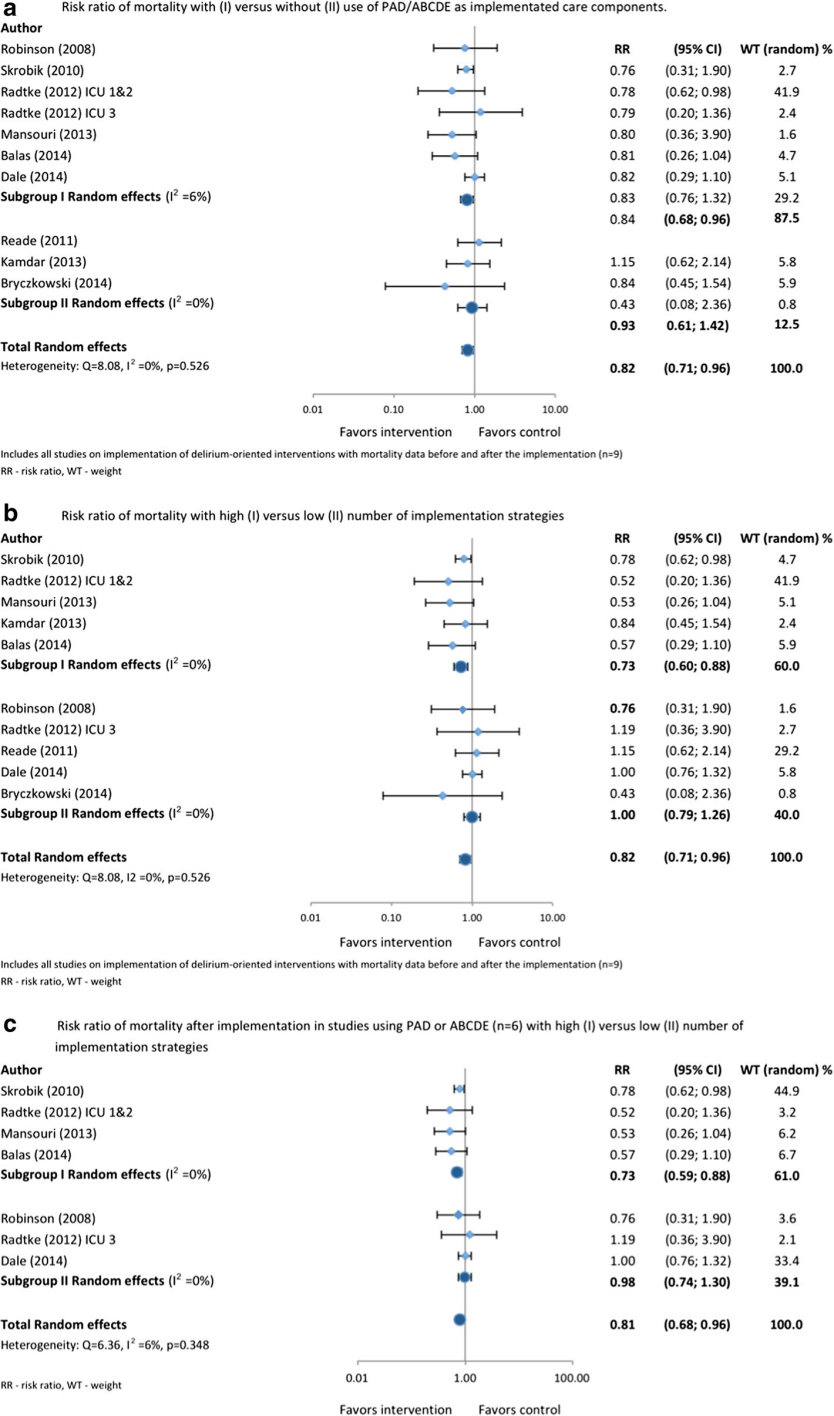
**Pooled analysis of determinants of changes in mortality (risk ratio) in implementation studies (n = 9) that included delirium-oriented interventions.** Determinants of mortality reduction that were studied were: use of either the guideline for the management of pain, agitation and delirium (PAD) or the awakening and breathing coordination, choice of sedative, delirium monitoring and management and early mobility (ABCDE) bundle **(a)** or use of high or low number of implementation strategies **(b)**. **(c)** Impact of high or low number of strategies within the studies reporting mortality and using PAD/ABCDE (n = 6). See text for more details.

Seven of the nine studies with mortality data before versus after implementation showed a reduction in mortality ranging from 2.9% to 12% (Table 3). Mortality was most often defined as hospital mortality (n = 6), but sometimes as ICU mortality [24,36] and 30-day mortality [20]. Three of these studies reported a statistically significant decrease in mortality between 6.5% (*P* = 0.009) and 12% (*P* = 0.046, Table 3) [16,20,24]. In the pooled analysis of all (n = 9) studies with mortality data, the mortality rates after implementation declined overall (RR = 0.82; 95% CI 0.71, 0.96 (Figure 3a). There was no inconsistency between the studies for this association (*I*^2^ = 0%, *P* = 0.526). Studies using PAD/ABCDE reported reduced mortality, whereas studies that did not use these frameworks did not (RR = 0.81; 95% CI 0.69, 0.96 versus RR = 0.93; 95% CI 0.61, 1.42). However, this difference in mortality risk reduction between the pooled data in studies with and without PAD/ABCDE did not reach statistical significance (*P* = 0.531). Mortality risk reduction was significantly higher (*P* = 0.0424) in studies that used high number of implementation strategies (RR = 0.73; 95% CI 0.60, 0.88) compared with studies with low number (Figure 3b). Further, in the studies that used the PAD guideline or ABCDE approach (n = 6) (Figure 3c) mortality reduction was higher (*P* = 0.0478) in studies that used a higher number of implementation strategies (RR = 0.73; 95% CI 0.59, 0.88 versus RR = 0.98; 95% CI 0.74, 1.30).

## Discussion

This systematic review and structured analysis of the literature aimed to summarize the implementation strategies and their effectiveness to change practice with regard to delirium assessment, prevention and management in the ICU and clinical outcomes. To accomplish this goal we tried to address both the why and the how questions regarding implementation. With regard to the why, an important finding of this review indicating that multi-component implementation that included delirium-oriented interventions in critically ill patients can be useful, is that many studies reported improvements of both process outcomes (delirium screening adherence, knowledge) and clinical outcomes (short-term mortality and ICU LOS). With regard to the how, several results of this review are worth highlighting: 1) some individual strategies such as audit and feedback and tailored interventions may be important to establish clinical outcome improvements, but otherwise robust data on effectiveness of specific implementation strategies are scarce; 2) using implementation strategies targeted not only at the health care professional but also at organizational, financial or regulatory domains is associated with better clinical outcomes; 3) using a higher number of implementation strategies (that is, six or more) concomitantly and delirium management being integrated according to the PAD guidelines or the ABCDE care bundle, are associated with positive effects of implementation efforts on clinical outcome, and 4) in contrast, a high number of implementation strategies and PAD/ABCDE use were not associated with reductions in delirium incidence. With regard to the third finding, it is imperative to note that the association between the use of six or more implementation strategies and mortality reductions should be regarded as a hypothesis-generating finding with regard to the effectiveness of implementation interventions for clinical outcome improvement, and therefore, does not imply that using more implementation strategies will definitely result in improved outcomes.

Our results seem to be consistent with the premises of the Society of Critical Care Medicine (SCCM) guideline on management of Pain, Agitation and Delirium (PAD) [3] and the ABCDE care bundle, that: 1) integrated management of pain, agitation/sedation and delirium together with early mobilization should be a component of the plan of ICUs to improve patient safety and comfort, and 2) complying with these components of evidence-based critical care has the potential to improve clinical outcomes depending on the baseline practices of any individual ICU and the patient population. Of the evidence-based interventions mentioned, early mobilization is the only intervention that has been shown to improve both delirium and clinical outcomes, but regrettably the integrated nature of both PAD and ABCDE precluded us from studying early mobilization implementation in isolation. Establishing such integrated management on a daily basis in all patients and by all ICU health care professionals is not an easy task, as it requires consideration of an intense amount of human factors and cultural adaptations. The data from this review support that putting effort into implementation may be worthwhile, while at the same time confirming that not all programs will meet with the same success. Importantly, we cannot exclude that the positive effects of using a high number of implementation interventions on mortality may in part be explained by the Hawthorne effect, meaning that using many implementation strategies at the same time may have improved quality of care due to improved attention for specific aspects of care, which may not always have been linked directly to delirium [38]. Another explanation may be that local ICU culture in these studies - which typically is unmeasured and thus unaccounted for - may have promoted successful implementation of changes into clinical practice. For instance, an ICU team consisting of professionals who are capable of adopting new practices within a limited time frame and that has acquired effective communication and collaboration across different types of health care professionals is probably more likely to implement multiple strategies successfully compared to a team that lacks these characteristics. The number of implementation strategies used may then confound the true causal association between local ICU culture and improved clinical outcome.

Although this review focused on delirium in the ICU, targeting delirium alone would not suffice to establish outcome improvements. Therefore, we argue that delirium screening alone would not likely establish mortality reduction when not embedded in an ABCDE bundle, for instance [7]. In other words, it is the circumstances leading to or sustaining brain dysfunction that should be dealt with in the first place. This view, that exclusively dealing with delirium may not suffice to improve clinical outcomes, is supported by a recent study showing that the attributable mortality caused by delirium in ICU patients is questionable and that long-term sequelae may be better clinical outcome measures for delirium-related outcomes than short-term mortality [39]. On the other hand, it is perceivable that delirium-focused management embedded in PAD or ABCDE may establish outcome improvement in spite of the fact that delirium may not be causally linked to mortality directly, analogous to lactate-guided management that may improve outcome in critically ill patients, in spite of lactate not being causally linked to mortality [40].

Several methodological limitations of this review need to be addressed. First, the included studies showed strong heterogeneity with regard to design, focus of implementation (prevention, assessment or management of delirium as primary focus or delirium-oriented interventions being part of the implementation program but not the main focus), applied implementation strategies and model and whether the study was primarily aimed at studying the implementation itself or not. Definitions of process and clinical outcomes varied between studies. For instance, delirium measures varied importantly between studies ranging from delirium incidence after admission to ICU to percentage of ICU days with delirium present per patient, which hampered comparability. Second, although early mobilization seems to be the only intervention within PAD/ABCDE that has been shown to affect both delirium and clinical outcomes, we could not isolate studies specifically reporting an implementation intervention that linked delirium and early mobilization implementation with clearly defined process or outcome measures, as per our inclusion criteria. Third, in spite of rigorous assessment of the implementation strategies that were used in included studies according to predefined EPOC definitions, a potential limitation hampering interpretation of the association between improved outcome and number of strategies is that the effort put in to execute these implementation strategies could not be assessed. For instance, two studies using the same number of strategies may still differ with regard to the efficacy of the implementation due to ongoing educational efforts in one but only a single educational session in the other study. We speculate that when more effort is put into the implementation it may be more successful even with the same number of implementation strategies used. Fourth, there is some evidence that suggests that uncontrolled pre-post test studies as included in this review may overestimate the effects of implementation or quality improvement studies [41]. Fifth, the results on ICU LOS should be considered cautiously because concurrent changes in mortality may affect ICU LOS, instead of the implementation intervention itself being responsible for lower ICU LOS, as censoring by death may bias and (theoretically) even reverse the associations found. On the other hand, strengths of this review include the systematic assessment of the implementation strategies by three independent investigators based on the description of strategies provided by EPOC, the focus on the clinical endpoints and the systematic assessment of methodological quality. Furthermore, inconsistency of the pooled analysis with regard to the clinical outcomes was low, which supports the generalizability of our findings.

Summarizing the current status of implementation work that has been done to date with regard to ICU delirium reveals which implementation strategies have not yet been studied extensively in this field. For example, reminders and computerized support have mostly been previously found to be effective strategies [11], whereas these strategies did not stand out in this review; assessment of these strategies in future work aimed at ICU delirium should therefore be considered. We think that our work may encourage health policy makers to invest in multifaceted implementation efforts to improve care for delirious ICU patients.

More research is necessary to elucidate which types of individual strategies and/or which combination of strategies used in implementation programs are most successful in establishing mortality reduction in delirious critically ill patients. Further, several aspects of implementation deserve further evaluation, as this review shows that these issues in implementation have lacked attention, such as cultural aspects pertaining to the medical ICU team, nurse-physician interaction and establishing sustainability of practice changes [12]. Prospective, adequately powered before-after studies may be most suitable for evaluation of practice changes and cluster-randomized trials are conceivably the best study designs to evaluate the effect of implementation strategies on outcome improvements [42]. Therefore, an important issue to be considered is the distinction between successful practice change and clinical outcome improvements in implementation research. In our study successful implementation was evident in most studies on delirium screening implementation that showed improved adherence, even without known benefit for clinical outcomes. On the other hand cumbersome implementation may result in improved outcomes.

Finally, detailed information on extent, form and content of implementation interventions, especially education, was often lacking in studies on implementation (data not shown). Therefore, reproducibility of delirium implementation research should also be taken into account in future investigations.

## Conclusion

This review and meta-analysis shows that multifaceted implementation programs that included assessment, prevention and management of ICU delirium have been shown to effectively change adherence to delirium screening and delirium knowledge. Implementation programs may enhance their effectiveness when not only health care professionals are targeted for behavioral change but also organizational changes are employed. Although using more rather than fewer implementation strategies simultaneously and delirium management being integrated with structured pain and agitation management (PAD), awakening and breathing coordination and early mobilization (ABCDE bundle) were associated with improved clinical outcomes, these results should be regarded as preliminary and hypothesis-generating with regard to the link between implementation practice and outcome improvement. Therefore, to determine whether these associations are causal our findings require confirmation and further study is needed on the most effective implementation strategies and the importance of focusing on delirium as an important form of organ failure within implementation programs aimed at practice change.

## Key messages

- Implementation programs can effectively improve delirium screening adherence or knowledge, but have had varying effects on delirium incidence and use of antipsychotic drugs.
- There seems to be no easy way out implementing delirium-oriented interventions, especially when combined with related care components as described in the PAD guidelines or ABCDE bundle: to implement these inclusive, integrated management frameworks, use of multiple implementation strategies concurrently that are targeted both at the care providers and at organizational aspects seems to be necessary.
- Successful implementation, meaning effective practice change, should be clearly delineated from the effect of such practice changes on clinical outcomes.
- Robust data on effectiveness of specific implementation strategies with regard to the care of delirious critically ill patients are scarce and there is a lack of data on the association between specific practice changes (for example, delirium screening) and improvements in clinical outcomes.

## Additional files

Additional file 1:
**The search strategy used in the systematic review.** This file provides details of the search strategy. Additional file 2:
**Adapted rating system from Anderson and Sharpe.** Explanation of the quality assessment tool that was used for included studies. Additional file 3:
**Quality rating.** Table showing quality assessment details of included implementation studies in the systematic review. Additional file 4:
**Pooled analysis of determinants of change in delirium incidence (risk ratio) in implementation studies (n = 8) that included delirium-oriented interventions.** of delirium incidence reduction that were studied were: use of either the guideline for the management of pain, agitation: and delirium (PAD) or the awakening and breathing coordination, choice of sedative, delirium monitoring and management, and early mobility (ABCDE) bundle **(a)** or use of high or low number of implementation strategies **(b)**. **(c)** Both studies that focused on delirium screening implementation and studies that did not (but, for example, implemented the ABCDE bundle), found no changes in delirium incidence after the implementation. Only two studies (van den Bogaard and Reade) on delirium screening implementation were included of which individual patient data could be retrieved from authors. See text for more details.

## Abbreviations

ABCDE bundle: awakening and breathing coordination, choice of sedative, delirium monitoring and management and early mobility bundle
EPOC: Cochrane Effective Practice and Organisation of Care group, classification system checklist
LOS: length of stay
PAD guideline: guideline for the management of pain, agitation and delirium
PRISMA: Preferred Reporting Items for Systematic Reviews and Meta-Analyses
RCT: randomized controlled trial
WMD: weighted mean difference

## References

[1] Engel GL, Romano J (1959). Delirium, a syndrome of cerebral insufficiency. J Chronic Dis.

[2] Hipp DM, Ely EW (2012). Pharmacological and nonpharmacological management of delirium in critically ill patients. Neurotherapeutics.

[3] Barr J, Fraser GL, Puntillo K, Ely EW, Gelinas C, Dasta JF (2013). Clinical practice guidelines for the management of pain, agitation, and delirium in adult patients in the intensive care unit. Crit Care Med.

[4] Vasilevskis EE, Pandharipande PP, Girard TD, Ely EW (2010). A screening, prevention, and restoration model for saving the injured brain in intensive care unit survivors. Crit Care Med.

[5] Girard TD, Kress JP, Fuchs BD, Thomason JW, Schweickert WD, Pun BT (2008). Efficacy and safety of a paired sedation and ventilator weaning protocol for mechanically ventilated patients in intensive care (Awakening and Breathing Controlled trial): a randomised controlled trial. Lancet.

[6] Schweickert WD, Pohlman MC, Pohlman AS, Nigos C, Pawlik AJ, Esbrook CL (2009). Early physical and occupational therapy in mechanically ventilated, critically ill patients: a randomised controlled trial. Lancet.

[7] van der Jagt M, Trogrlic Z, Ista E (2014). Untangling ICU delirium: is establishing its prevention in high-risk patients the final frontier?. Intensive Care Med.

[8] Liberati A, Altman DG, Tetzlaff J, Mulrow C, Gotzsche PC, Ioannidis JP (2009). The PRISMA statement for reporting systematic reviews and meta-analyses of studies that evaluate health care interventions: explanation and elaboration. PLoS Med.

[9] Slomka J, Hoffman-Hogg L, Mion LC, Bair N, Bobek MB, Arroliga AC (2000). Influence of clinicians’ values and perceptions on use of clinical practice guidelines for sedation and neuromuscular blockade in patients receiving mechanical ventilation. Am J Crit Care.

[10] EPOC:2002. http://epoc.cochrane.org/sites/epoc.cochrane.org/files/uploads/datacollectionchecklist.pdf.

[11] Grol R, Grimshaw J (2003). From best evidence to best practice: effective implementation of change in patients’ care. Lancet.

[12] Sinuff T, Muscedere J, Adhikari NK, Stelfox HT, Dodek P, Heyland DK (2013). Knowledge translation interventions for critically ill patients: a systematic review*. Crit Care Med.

[13] Anderson LA, Sharpe PA (1991). Improving patient and provider communication: a synthesis and review of communication interventions. Patient Educ Couns.

[14] DerSimonian R, Laird N (1986). Meta-analysis in clinical trials. Control Clin Trials.

[15] Higgins JP, Thompson SG, Deeks JJ, Altman DG (2003). Measuring inconsistency in meta-analyses. BMJ.

[16] Balas MC, Vasilevskis EE, Olsen KM, Schmid KK, Shostrom V, Cohen MZ (2014). Effectiveness and safety of the awakening and breathing coordination, delirium monitoring/management, and early exercise/mobility bundle. Crit Care Med.

[17] van den Boogaard M, Pickkers P, van der Hoeven H, Roodbol G, van Achterberg T, Schoonhoven L (2009). Implementation of a delirium assessment tool in the ICU can influence haloperidol use. Crit Care.

[18] Riekerk B, Pen EJ, Hofhuis JG, Rommes JH, Schultz MJ, Spronk PE (2009). Limitations and practicalities of CAM-ICU implementation, a delirium scoring system, in a Dutch intensive care unit. Intensive Crit Care Nurs.

[19] Hager DN, Dinglas VD, Subhas S, Rowden AM, Neufeld KJ, Bienvenu OJ (2013). Reducing deep sedation and delirium in acute lung injury patients: a quality improvement project. Crit Care Med.

[20] Skrobik Y, Ahern S, Leblanc M, Marquis F, Awissi DK, Kavanagh BP (2010). Protocolized intensive care unit management of analgesia, sedation, and delirium improves analgesia and subsyndromal delirium rates. Anesth Analg.

[21] Bowen CM, Stanton M, Manno M (2012). Using Diffusion of Innovations Theory to implement the confusion assessment method for the intensive care unit. J Nurs Care Qual.

[22] Soja SL, Pandharipande PP, Fleming SB, Cotton BA, Miller LR, Weaver SG (2008). Implementation, reliability testing, and compliance monitoring of the Confusion Assessment Method for the Intensive Care Unit in trauma patients. Intensive Care Med.

[23] Gesin G, Russell BB, Lin AP, Norton HJ, Evans SL, Devlin JW (2012). Impact of a delirium screening tool and multifaceted education on nurses’ knowledge of delirium and ability to evaluate it correctly. Am J Crit Care.

[24] Mansouri P, Javadpour S, Zand F, Ghodsbin F, Sabetian G, Masjedi M (2013). Implementation of a protocol for integrated management of pain, agitation, and delirium can improve clinical outcomes in the intensive care unit: a randomized clinical trial. J Crit Care.

[25] Pun BT, Gordon SM, Peterson JF, Shintani AK, Jackson JC, Foss J (2005). Large-scale implementation of sedation and delirium monitoring in the intensive care unit: a report from two medical centers. Crit Care Med.

[26] Radtke FM, Heymann A, Franck M, Maechler F, Drews T, Luetz A (2012). How to implement monitoring tools for sedation, pain and delirium in the intensive care unit: an experimental cohort study. Intensive Care Med.

[27] Eastwood GM, Peck L, Bellomo R, Baldwin I, Reade MC (2012). A questionnaire survey of critical care nurses’ attitudes to delirium assessment before and after introduction of the CAM-ICU. Aust Crit Care.

[28] Kamdar BB, King LM, Collop NA, Sakamuri S, Colantuoni E, Neufeld KJ (2013). The effect of a quality improvement intervention on perceived sleep quality and cognition in a medical ICU. Crit Care Med.

[29] Scott P, McIlveney F, Mallice M (2013). Implementation of a validated delirium assessment tool in critically ill adults. Intensive Crit Care Nurs.

[30] Dale CR, Kannas DA, Fan VS, Daniel SL, Deem S, Yanez ND (2014). Improved analgesia, sedation, and delirium protocol associated with decreased duration of delirium and mechanical ventilation. Ann Am Thorac Soc.

[31] Kastrup M, Nolting MJ, Ahlborn R, Braun JP, Grubitzsch H, Wernecke KD (2011). An electronic tool for visual feedback to monitor the adherence to quality indicators in intensive care medicine. J Int Med Res.

[32] Robinson BR, Mueller EW, Henson K, Branson RD, Barsoum S, Tsuei BJ (2008). An analgesia-delirium-sedation protocol for critically ill trauma patients reduces ventilator days and hospital length of stay. J Trauma.

[33] Devlin JW, Fong JJ, Howard EP, Skrobik Y, McCoy N, Yasuda C (2008). Assessment of delirium in the intensive care unit: nursing practices and perceptions. Am J Crit Care.

[34] Page VJ, Navarange S, Gama S, McAuley DF (2009). Routine delirium monitoring in a UK critical care unit. Crit Care.

[35] Reade MC, Eastwood GM, Peck L, Bellomo R, Baldwin I (2011). Routine use of the Confusion Assessment Method for the Intensive Care Unit (CAM-ICU) by bedside nurses may underdiagnose delirium. Crit Care Resusc.

[36] Bryczkowski SB, Lopreiato MC, Yonclas PP, Sacca JJ, Mosenthal AC (2014). Delirium prevention program in the surgical intensive care unit improved the outcomes of older adults. J Surg Res.

[37] Khalifezadeh A, Safazadeh S, Mehrabi T, Mansour BA (2011). Reviewing the effect of nursing interventions on delirious patients admitted to intensive care unit of neurosurgery ward in Al-Zahra Hospital, Isfahan University of Medical Sciences. Iran J Nurs Midwifery Res.

[38] van Zanten AR, Brinkman S, Arbous MS, Abu-Hanna A, Levy MM, de Keizer NF (2014). Guideline bundles adherence and mortality in severe sepsis and septic shock. Crit Care Med.

[39] Klein Klouwenberg PM, Zaal IJ, Spitoni C, Ong DS, van der Kooi AW, Bonten MJ (2014). The attributable mortality of delirium in critically ill patients: prospective cohort study. BMJ.

[40] Jansen TC, van Bommel J, Schoonderbeek FJ, Sleeswijk Visser SJ, van der Klooster JM, Lima AP (2010). Early lactate-guided therapy in intensive care unit patients: a multicenter, open-label, randomized controlled trial. Am J Respir Crit Care Med.

[41] Eccles M, Grimshaw J, Campbell M, Ramsay C (2003). Research designs for studies evaluating the effectiveness of change and improvement strategies. Qual Saf Health Care.

[42] Ista E, Trogrlic Z, Bakker J, Osse R, van Achterberg T, van der Jagt M (2014). Improvement of care for ICU patients with delirium by early screening and treatment: study protocol of iDECePTIvE study. Implement Sci.

