# Supplementary material for: A systematic review of implementation strategies for assessment, prevention, and management of ICU delirium and their effect on clinical outcomes
Source: Crit Care. 2015 Apr 9;19(1):157. doi: 10.1186/s13054-015-0886-9 (PMC4428250; doi:10.1186/s13054-015-0886-9)
Supplement: Additional file 1: — The search strategy used in the systematic review. This file provides details of the search strategy. [file 13054_2015_886_MOESM1_ESM.pdf]

Additional file 1: Search string and search results

Database	Search string	Identified	Duplicates excluded	Retrieved
**PubMed**	(Deliri*[tw] OR Confusion*[tiab] OR Psychosis[tiab] OR Psychoses[tiab] OR Psychotic[tw] OR brain failure*[tiab]) AND (Intensive Care*[tw] OR ICU*[tiab] OR critical care*[tiab] OR critically ill*[tiab])	1917	15	(1902)
**Embase**	('intensive care psychosis'/de OR ((Deliri* OR Psychotic OR Confusion* OR Psychosis OR Psychoses) NEAR/6 ('Intensive Care' OR ICU OR 'critical care' OR 'critically ill' OR 'critical illness')):de,ab,ti)	1232	904	(328)
**PsycINFO**	((Deliri* OR Psychotic OR Confusion* OR Psychosis OR Psychoses) ADJ6 ('Intensive Care' OR ICU OR 'critical care' OR 'critically ill' OR 'critical illness'))	181	125	(56)
**Cochrane**	((Deliri* OR Psychotic OR Confusion* OR Psychosis OR Psychoses) NEAR/6 ('Intensive Care' OR ICU OR 'critical care' OR 'critically ill' OR 'critical illness'))	15	8	(7)
**CINAHL**	MM "ICU Psychosis" OR SU((Deliri* OR Confusion* OR Psychosis OR Psychoses OR Psychotic OR "brain failure*") AND ("Intensive Care*" OR ICU* OR "critical care*" OR "critically ill*"))	636	447	(189)
**Total:**		3981	1499	2482
**Total # of studies excluded before year 2000**				514
**Remaining # of identified studies**				**1968**
