# Supplementary material for: A systematic review of implementation strategies for assessment, prevention, and management of ICU delirium and their effect on clinical outcomes
Source: Crit Care. 2015 Apr 9;19(1):157. doi: 10.1186/s13054-015-0886-9 (PMC4428250; doi:10.1186/s13054-015-0886-9)
Supplement: Additional file 2: — Adapted rating system from Anderson and Sharpe. Explanation of the quality assessment tool that was used for included studies. [file 13054_2015_886_MOESM2_ESM.pdf]

Additional file 2: Adapted Rating system from Anderson and Sharpe

**Design of study or assignment rating**	**Rating**
Experimental: RCT, random allocation; CCT, quasi-random allocation; three data collection points before and after the intervention	1
Quasi-experimental: CBA, comparable control sites	1
Quasi-experimental: nonequivalent control sites	0
Single group before-after tests with baseline measurement	0
**Content**	
Intervention, implementation strategy is clearly described	1
**Sample size**	
Described and justified. An n per group sufficient to detect a significant effect ($p<0.05$) with a power of 0.80 or reported Power calculation	1
**Validity and reliability of instruments**	
Unobtrusive observations, rater procedure described and $r>0.80$	2
Unobtrusive observations, rater procedure not described or $r<0.80$	1
Obtrusive observations, rater procedure not described or $r<0.80$	0
**Test statistics**	
Test statistics are described	1
**Significance**	
p value or confidence interval is given	1

CBA=controlled before-and-after study, CCT=controlled clinical trial, ITS=interrupted time series.
