# Supplementary material for: A systematic review of implementation strategies for assessment, prevention, and management of ICU delirium and their effect on clinical outcomes
Source: Crit Care. 2015 Apr 9;19(1):157. doi: 10.1186/s13054-015-0886-9 (PMC4428250; doi:10.1186/s13054-015-0886-9)
Supplement: Additional file 3: — Quality rating. Table showing quality assessment details of included implementation studies in the systematic review. [file 13054_2015_886_MOESM3_ESM.pdf]

Additional file 3: Quality Rating of Implementation Studies

Author	Design of study or assignment rating	Content	Sample size	Validity and reliability of instruments	Test statistics	Significance	Total
Balas et al, 2014, USA	1	1	0	2	1	1	**6**
Bowen et al, 2012, USA	0	1	0	2	1	0	**4**
Devlin et al, 2008, USA	1	1	1	2	1	1	**7**
Eastwood et al, 2012, Australia	0	1	0	2	1	1	**5**
Gesin et al, 2012, USA	1	1	1	2	1	1	**7**
Hagar et al, 213, USA	1	1	0	2	1	1	**6**
Kamdar, et al, 2013, USA	1	1	1	2	1	1	**7**
Kastrup et al, 2011, Germany	1	1	0	2	1	1	**6**
*Khalifezadeh et al, 2011, Iran	1	0	0	0	1	0	**2**
Mansouri et al, 2013, Iran	1	1	0	1	1	1	**5**
Page et al, 2009, UK	0	1	0	2	1	1	**5**
Pun et al, 2005, USA	1	1	0	2	1	1	**6**
Radtke , Heymann et al, 2012, Germany	1	1	0	2	1	1	**6**
Reade et al, 2011, Australia	0	1	0	2	1	1	**5**
Riekerk et al, 2009, The Netherlands	1	1	0	2	1	1	**6**
Robinson et al, 2008, USA	1	1	0	2	1	1	**6**
Scott et al, 2012, UK	1	1	0	2	0	0	**4**
Skrobik et al, 2010, Canada	1	1	0	2	1	1	**6**
Soja et al, 2008, USA	1	1	0	2	1	1	**6**
Van den Boogaard et al, 2009, The Netherlans	1	1	0	2	1	1	**6**
Dale et al, 2014, USA	1	1	0	2	1	1	**6**
Bryczkowski et al, 2014, USA	1	1	0	2	1	1	**6**

The item validity and reliability was adapted adding one extra point for first option (Unobtrusive observations, rater procedure described and r >0.80) for a better distinction when implementation procedure is well described.
* Excluded
