# Supplementary figures and images for: A systematic review of implementation strategies for assessment, prevention, and management of ICU delirium and their effect on clinical outcomes
Source: Crit Care. 2015 Apr 9;19(1):157. doi: 10.1186/s13054-015-0886-9 (PMC4428250; doi:10.1186/s13054-015-0886-9)

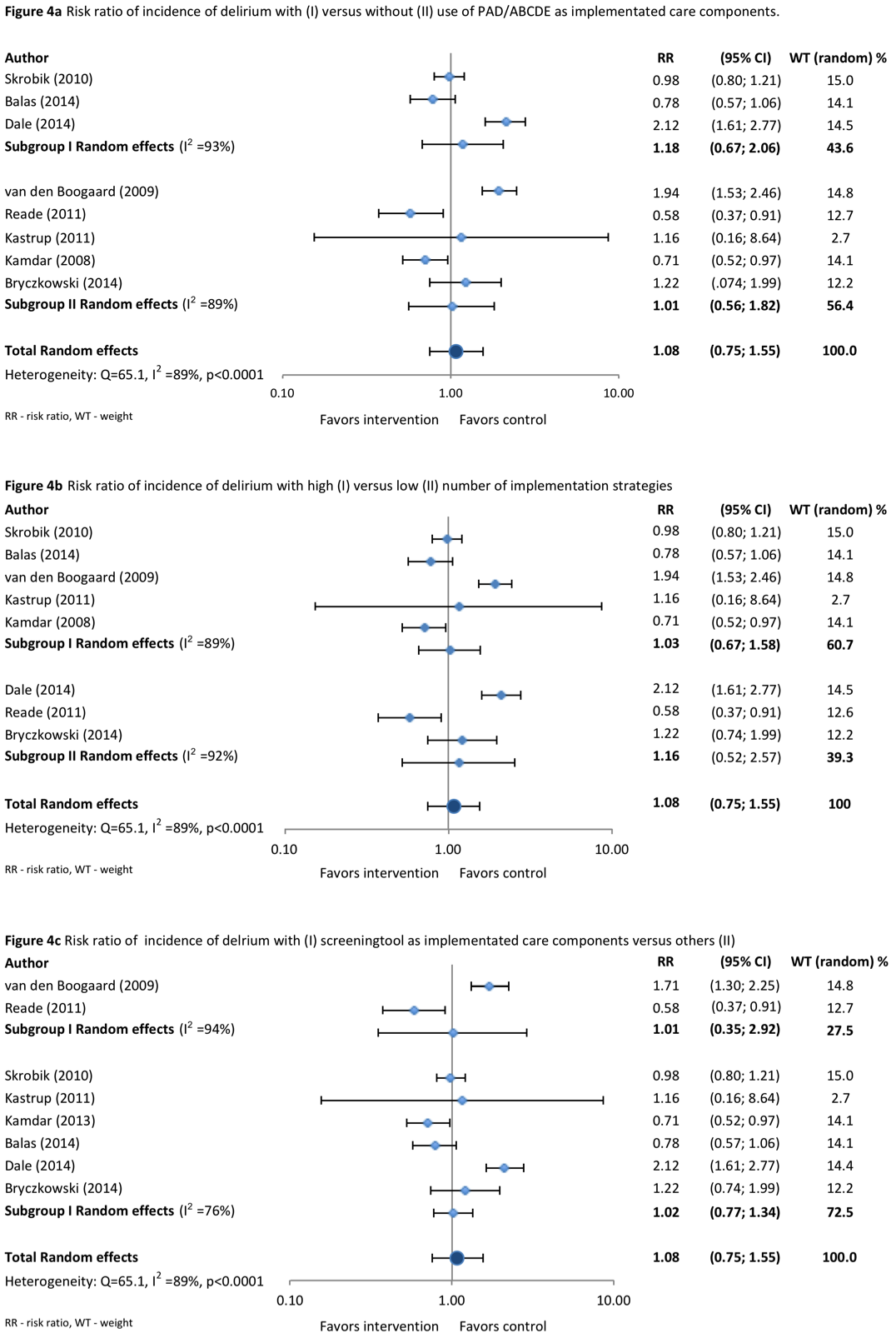

Supplement: Additional file 4: — Pooled analysis of determinants of change in delirium incidence (risk ratio) in implementation studies (n = 8) that included delirium-oriented interventions. of delirium incidence reduction that were studied were: use of either the guideline for the management of pain, agitation: and delirium (PAD) or the awakening and breathing coordination, choice of sedative, delirium monitoring and management, and early mobility (ABCDE) bundle (a) or use of high or low number of implementation strategies (b). (c) Both studies that focused on delirium screening implementation and studies that did not (but, for example, implemented the ABCDE bundle), found no changes in delirium incidence after the implementation. Only two studies (van den Bogaard and Reade) on delirium screening implementation were included of which individual patient data could be retrieved from authors. See text for more details. [file 13054_2015_886_MOESM4_ESM.png]
